# Effects of aggregation of drug and diagnostic codes on the performance of the high-dimensional propensity score algorithm: an empirical example

**DOI:** 10.1186/1471-2288-13-142

**Published:** 2013-11-19

**Authors:** Hoa V Le, Charles Poole, M Alan Brookhart, Victor J Schoenbach, Kathleen J Beach, J Bradley Layton, Til Stürmer

**Affiliations:** 1 Department of Epidemiology, University of North Carolina at Chapel Hill, Chapel Hill, USA; 2 GlaxoSmithKline, Research Triangle Park, Durham, North Carolina, USA

**Keywords:** Aggregation, Anatomical therapeutic chemical classification, Clinical classification software, Confounding by indication, Infrequent exposure, Propensity score, Small sample, Rare outcome

## Abstract

**Background:**

The High-Dimensional Propensity Score (hd-PS) algorithm can select and adjust for baseline confounders of treatment-outcome associations in pharmacoepidemiologic studies that use healthcare claims data. How hd-PS performance is affected by aggregating medications or medical diagnoses has not been assessed.

**Methods:**

We evaluated the effects of aggregating medications or diagnoses on hd-PS performance in an empirical example using resampled cohorts with small sample size, rare outcome incidence, or low exposure prevalence. In a cohort study comparing the risk of upper gastrointestinal complications in celecoxib or traditional NSAIDs (diclofenac, ibuprofen) initiators with rheumatoid arthritis and osteoarthritis, we (1) aggregated medications and International Classification of Diseases-9 (ICD-9) diagnoses into hierarchies of the Anatomical Therapeutic Chemical classification (ATC) and the Clinical Classification Software (CCS), respectively, and (2) sampled the full cohort using techniques validated by simulations to create 9,600 samples to compare 16 aggregation scenarios across 50% and 20% samples with varying outcome incidence and exposure prevalence. We applied hd-PS to estimate relative risks (RR) using 5 dimensions, predefined confounders, ≤ 500 hd-PS covariates, and propensity score deciles. For each scenario, we calculated: (1) the geometric mean RR; (2) the difference between the scenario mean ln(RR) and the ln(RR) from published randomized controlled trials (RCT); and (3) the proportional difference in the degree of estimated confounding between that scenario and the base scenario (no aggregation).

**Results:**

Compared with the base scenario, aggregations of medications into ATC level 4 alone or in combination with aggregation of diagnoses into CCS level 1 improved the hd-PS confounding adjustment in most scenarios, reducing residual confounding compared with the RCT findings by up to 19%.

**Conclusions:**

Aggregation of codes using hierarchical coding systems may improve the performance of the hd-PS to control for confounders. The balance of advantages and disadvantages of aggregation is likely to vary across research settings.

## Background

Although early detection and assessment of drug safety signals are important [1-3], post-approval drug safety studies often face challenges such as small size, rare incidence of adverse outcomes, and low exposure prevalence after the launch of a new drug. In addition, nonrandomized studies of treatment effects in healthcare data are vulnerable to confounding bias. Propensity Score (PS) methods are increasingly used to control for measured potential confounders, especially in pharmacoepidemiologic studies of rare outcomes in the presence of many covariates from different data dimensions of administrative healthcare databases [4-7]. Methods of selecting variables for PS models based on substantive knowledge have been proposed [8-12], but substantive knowledge may often be lacking, and the meaning of various medical codes may often be unclear [13]: Seeger et al. proposed that health care claims may serve as proxies in hard-to-predict ways for important unmeasured covariates [14]; Stürmer et al. used PS models with over 70 variables representing medical codes present during a baseline period [5]; Johannes et al. created a PS model that considered as candidate variables the 100 most frequently occurring diagnoses, procedures, and outpatient medications in healthcare claims [15]. A recently-developed strategy for selecting variables from a large pool of baseline covariates for PS analyses is the use of computer-applied algorithms [16,17], such as the High-Dimensional Propensity Score (hd-PS) algorithm. The hd-PS automatically defines and selects variables for inclusion in the PS estimating model to adjust treatment effect estimates in studies using automated healthcare data [16,18].

The hd-PS algorithm prioritizes variables within each data dimension (e.g., inpatient diagnoses, inpatient procedures, outpatient diagnoses, outpatient procedures, dispensed prescription drugs) by their potential for confounding control based on their prevalence and on bivariate associations with the treatment and with the study outcome [16,19]. Version 1 of the hd-PS algorithm excludes variables found in fewer than 100 patients (exposed and unexposed combined) and variables with zero/undefined covariate-exposure association or zero/undefined covariate-outcome association. Once variables have been prioritized, a predefined number of variables with the highest potential for confounding per dimension is chosen to be included in the PS.

Combining medications or medical diagnoses into higher-level groupings increases the prevalence of the aggregated covariate which may increase the chances of a variable being selected by the algorithm. However, aggregation may also weaken covariate-exposure and/or covariate-outcome relations and reduce variable prioritization in the Bross formula [19]. In addition to the selection issue, control for a selected aggregated variable may lead to residual confounding in the adjusted risk ratios if not all of its components have the same confounding effect. No study to date has assessed how hd-PS performance is affected by aggregating medications and/or medical diagnoses, especially in cohorts with relatively few patients, rare outcome incidence, or low exposure prevalence. To investigate the impact of aggregation on hd-PS performance in cohorts with low outcome incidence or exposure prevalence, we created an empirical example based on prior research [16,20] with an observed elevated crude risk ratio, likely due to confounding by indication in studies of upper gastrointestinal (UGI) complications in rheumatoid arthritis (RA) or osteoarthritis (OA) patients initiating celecoxib compared to traditional non-steroidal anti-inflammatory agents (tNSAIDs). Celecoxib has been shown to decrease the risk of UGI complications in several randomized controlled trials (RCT) by approximately 50% [21-26]. We therefore assume that a treatment effect estimate closer to 0.50 is less biased by confounding.

## Methods

### Selection of the study cohort

We constructed an incident user cohort [27] to examine UGI complication in RA and OA patients initiating celecoxib or a tNSAID, specifically ibuprofen or diclofenac. All individuals with a first dispensing between 1 July 2003 and 30 September 2004 of celecoxib, ibuprofen, or diclofenac were drawn from the Truven Health Analytics MarketScan® Commercial Claims and Encounters [28]. MarketScan is a longitudinal healthcare claims database which captures patient demographics, inpatient and outpatient diagnoses and procedures, and medications from a selection of large private employers, health plans, government agencies and other public organizations. We selected patients who were age 18–65 years, belonged to a health insurance plan with full medical and pharmacy benefits, and had at least 6 months of enrollment history as of the date of first dispensing of a study or referent drug (the “index date”). During the 6 months prior to the index date, patients must have had a diagnosis of RA (ICD-9 code 714.x) or OA (ICD-9 code 715.x, 721.x) but no NSAID dispensing (including aspirin); and no record of gastrointestinal ulcer disorders, gastrointestinal hemorrhage, active renal, hepatic, coagulation disorders, allergies, malignancy, esophageal or gastroduodenal ulceration.

The study outcome — UGI complication — was defined as an inpatient or outpatient diagnosis for either first peptic ulcer disease complications including perforation, an UGI hemorrhage (ICD-9 code 531.x, 532.x, 533.x, 534.x, 535.x, 578.0), or a physician service code for UGI hemorrhage (Current Procedure Terminology (CPT) code 43255 or ICD-9 procedure code 44.43). The complication must have occurred during the 60 days after initiation of the study drug. These outcome definitions have been validated for 1,762 patients in an independent hospital discharge database with a positive predictive value of 90% against medical chart review [29].

### Aggregations of medications and medical diagnoses

Major U.S. administrative databases contain prescription medication information coded with non-hierarchical National Drug Codes (NDC) and generic drug names. The medications can be aggregated using the hierarchical Anatomical Therapeutic Chemical (ATC) drug classification developed by the World Health Organization (WHO) for drug utilization studies [30]. Similarly, medical diagnoses are represented by International Classification of Diseases, 9th Revision, Clinical Modification (ICD-9) codes. ICD-9 has limited hierarchical relationships [31], but the Clinical Classification Software (CCS) developed by the Agency for Healthcare Research and Quality (AHRQ) can be used to aggregate diagnoses into clinically meaningful groupings [32].

We aggregated medications to five levels of the ATC classification [30]. This system classifies active substances into different groups based on their target organ or system and their therapeutic, pharmacological and chemical properties. Drugs are classified into fourteen main groups (1^st^ level) with pharmacological or therapeutic subgroups (2^nd^ level). The 3^rd^ and 4^th^ levels are chemical, pharmacological or therapeutic subgroups, and the 5^th^ level is the chemical substance. Several ATC groups are subdivided into both chemical and pharmacological groups. The pharmacological group is often chosen if a new substance fits in both a chemical and pharmacological 4^th^ level. Substances in the same 4^th^ ATC level are not pharmacotherapeutically equivalent, as they may have different modes of action, therapeutic effects, drug interactions and adverse drug reaction profiles. New 4^th^ levels are commonly established if at least two approved substances fit in the group. A new substance not clearly belonging to any existing group of related substances of ATC 4^th^ level will often be placed in an X group (“other” group). There are very few new substances in each of the ATC 4^th^ levels with the same ATC 3^rd^ level for each ATC release. New substances belonging to different ATC 3^rd^ levels will have different codes for “X” groups in ATC 4^th^ level. Therefore, the mixed bag of “X” group is not an issue for the hd-PS algorithm.

We created several scenarios of code aggregation using the CCS groupings. In the base scenario, we applied the hd-PS with up to 5-digit granularity of ICD-9 for inpatient and outpatient diagnoses. Note that 3-digit ICD-9 codes are kept separate from 4- and 5-digit codes in the hd-PS despite the limited hierarchy between these levels. We transformed ICD-9 diagnoses into four-level CCS groupings via the cross-mapped ICD-9 to CCS multi-level diagnoses table [32]. There are 18, 134, 355 and 207 groupings in CCS levels 1, 2, 3 and 4, respectively. However, not all ICD-9 codes have a corresponding CCS code in all four levels. Therefore we created a “universal” CCS by using the most granular code available for each ICD-9 diagnosis code to obtain 355 groupings in CCS level 4. We separately investigated different levels of ICD-9 granularity by using the first 3- or 4-digit ICD-9 codes [16].

### Sampling techniques to generate conditions of different size, outcome incidence and exposure prevalence

**Figure 1:**
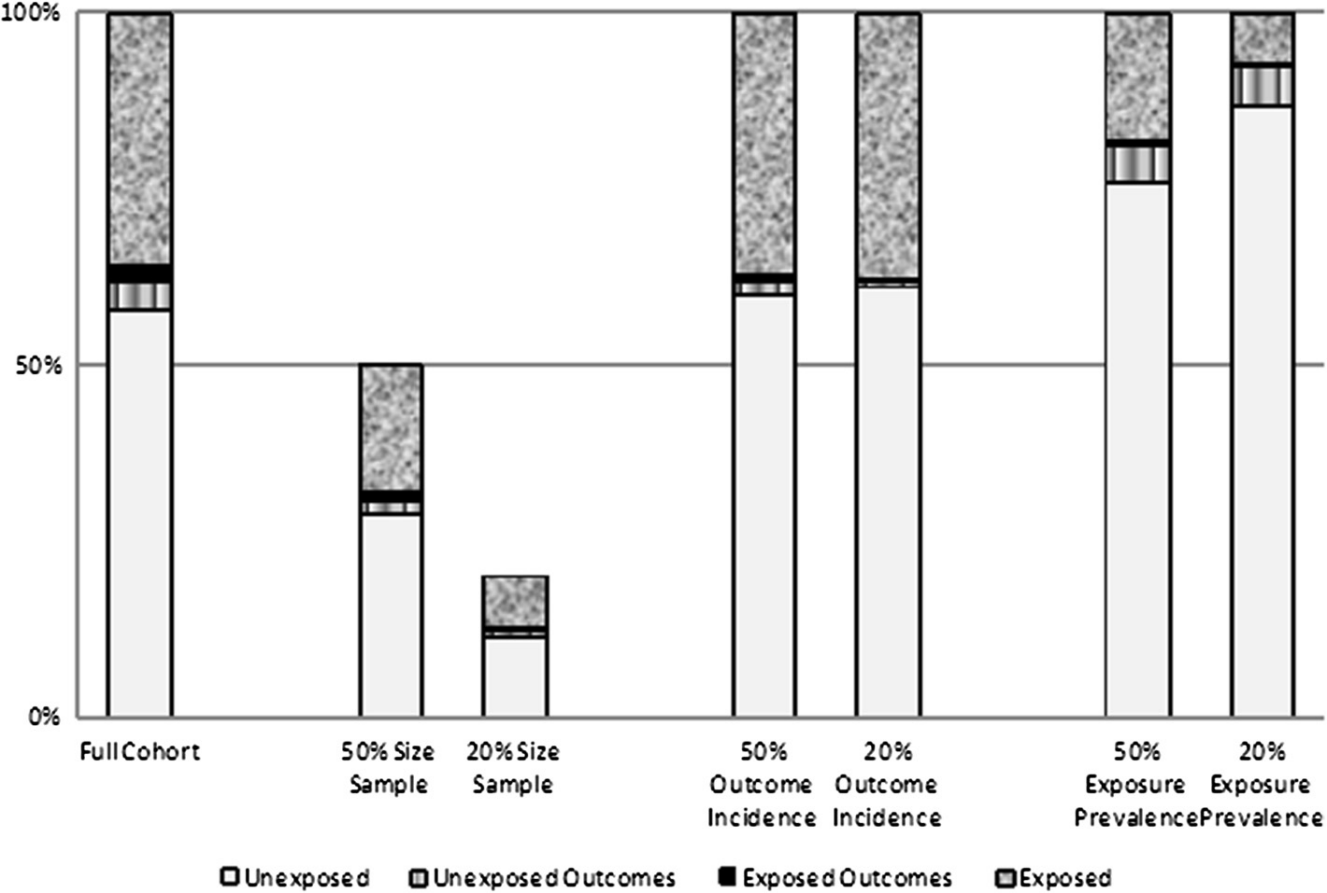
A visualization of the sampling techniques to generate 6 conditions of different size, outcome incidence and exposure prevalence from the full cohort.

The full cohort consisted of 18,829 patients (7,197 prescribed celecoxib and 11,632 prescribed ibuprofen or diclofenac); 117 patients developed an UGI complication. For each aggregation scenario (including no aggregation), we created six conditions of 100 sampled cohorts. First, we varied the total size of the cohort by drawing simple random samples of 50% (condition 1) and 20% (condition 2), 100 times each, without replacement. Second, we varied the outcome incidence by retaining all noncases, but drawing 50% (condition 3) and 20% (condition 4) simple random samples without replacement, 100 times each, from the 117 cases and re-coding the unselected cases as noncases. Finally, we varied the exposure prevalence by retaining all unexposed patients, but drawing 50% (condition 5) and 20% (condition 6) simple random samples, 100 times each, without replacement, from the exposed subjects and replacing the unselected exposed subjects with the same number of randomly selected unexposed patients (Figure 1).

### The hd-PS algorithm

We implemented the hd-PS algorithm with five data dimensions commonly available in automated healthcare databases: pharmacy claims; outpatient diagnoses; outpatient procedures; inpatient diagnoses; and inpatient procedures. The algorithm identifies the top 200 most prevalent covariates within each data dimension by creating binary variables for each diagnosis, procedure and medication. The prevalence of each variable depends on the granularity of the coding: the more aggregation, the higher the prevalence tends to be as subcodes are combined into aggregated codes. Each variable is assessed for 3 levels of its within-patient occurrence: (1) once; (2) sporadic (≥ median number of times); or (3) frequent (≥ 75^th^ percentile number of times) with details described elsewhere [16]. With the default setting of 200 variables for each dimension, 3,000 indicator variables (200 × 3 levels × 5 dimensions) are then prioritized according to their potential for confounding control based on their prevalence and bivariate associations with the treatment and with the study outcome according to the Bross formula [19]. By default, the top 500 indicator variables are selected for the PS.

### Statistical analysis

The hd-PS algorithm can augment the automatically-selected covariates with predefined covariates chosen by the investigator. For each condition-scenario combination, we fit 5 log binomial models: (1) an unadjusted crude model; (2) adjusted for basic covariates (age [continuous], gender, calendar year of drug initiation); (3) adjusted for basic plus extended, pre-selected covariates (hypertension, congestive heart failure, coronary artery disease, inflammatory bowel disease, prior dispensing of gastroprotective drugs, warfarin, antiplatelet drugs, and oral steroids) selected based on biological rationale in the literature [16,18,20,33-35]; (4) adjusted for basic plus hd-PS selected covariates; and (5) adjusted for basic, extended, and hd-PS selected covariates. The hd-PS was adjusted for by including indicator variables for each decile of PS in the regression model.

In the base scenario, we used generic drugs and up to 5-digit granularity of ICD-9, CPT or Healthcare Common Procedure Coding System (HCPCS). We then re-fitted all models in six scenarios for aggregation of medications, eight scenarios for aggregation of diagnoses, and one scenario that combined the medication and diagnosis aggregations that appeared to perform best across the six conditions of cohort samples.

We applied hd-PS to the full study cohort to estimate the treatment effect and used it as the reference value for comparison with results from the generated cohort conditions. For the 100 samples in each of the cohort conditions, we calculated summary statistics for the estimated risk ratios (geometric mean, 25^th^ and 75^th^ percentiles), including: the mean percentage of covariates selected by hd-PS in the full cohort that were also selected by hd-PS in the samples; the median number of exposed and unexposed subjects; the median number of exposed and unexposed outcomes. We evaluated each aggregation scenario by estimating the amount of residual confounding, calculated as the difference in the natural logarithms of the estimated risk ratio and the natural logarithm of 0.50, representing the RCT findings [21-26]. To estimate the change in residual confounding resulting from each aggregation scenario, we calculated the proportional difference in absolute degree of estimated confounding between the scenario of interest and the base (no aggregation) scenario. For example, for the 20% exposure prevalence cohorts (condition 6), the unadjusted (confounded but otherwise presumptively unbiased) estimate is RR_u_ = 0.97, and two confounded (but otherwise presumptively unbiased) estimates are RR_c1_ = 0.89 (base: basic, extended and hd-PS covariates; no aggregation) and RR_c2_ = 0.81 (combined diagnostic and medication aggregation). Assuming that the unconfounded (true) value is RR_t_ = 0.50, estimated confounding in the base estimate = │ln(0.89) – ln(0.50)│ =0.577; estimated confounding in the combined aggregation estimate = │ln(0.81) – ln(0.50)│ = 0.482. Thus, the proportional difference in absolute degree of estimated confounding between the two estimates = (0.482 - 0.577)/0.577 = −16.3%. We would conclude that the combined aggregation estimate is 16.3% less confounded than the base estimate.

Because of limited data availability, and to mimic as closely as possible the intention-to-treat analyses in the trials, we used a single prescription reimbursement claim as the treatment measure. The current study was exempt by the Institutional Review Board of University of North Carolina at Chapel Hill.

## Results

In the full cohort, there were 7,197 (38%) celecoxib and 11,632 (62%) ibuprofen or diclofenac initiators with 46 and 71 UGI events, respectively. Celecoxib users were older and had more risk factors for UGI complications than did the tNSAIDs users (Table 1). The RR for UGI complication associated with celecoxib versus tNSAIDs was 1.05 (95% CI: 0.72-1.52) in the crude model, compared to 0.92 (95% CI: 0.62-1.37) in the model that used hd-PS automated variable selection in addition to the basic covariates (Table 2). Consistent with the sampling procedures described above, the median numbers of patients in cohorts in conditions 1 and 2 were about 3,594 and 1,441, respectively; the median outcome incidence proportions in conditions 3 and 4 were about 0.3% and 0.1%, respectively, and the median exposure prevalence in conditions 5 and 6 were about 19% and 8%, respectively.

**Table 1:** **Characteristics of initiators of celecoxib or NSAIDs** (**ibuprofen or diclofenac**) **in a cohort 18**–**65 years old between 1 July 2003 and 30 September 2004 in the MarketScan database**: **age at the date of the first medication use and co**-**morbidities**/**use of medications as defined during six months prior to the first medication use**

**Characteristics**	**Celecoxib ** **n = 7,197 (38%)**		**Ibuprofen or Diclofenac ** **n = 11,632 (62%)**	
	**n**	**%**	**n**	**%**
Age (years)				
Median	56.0		52.0	
Mean	54.1		50.4	
Standard deviation	8.2		9.7	
18-35	235	3.3	996	8.6
36-45	854	11.9	2,164	18.6
46-55	2,373	33.0	4,339	37.3
56-65	3,735	51.9	4,133	35.5
Female	4,387	61.0	6,869	59.1
Hypertension	1,748	24.3	2,191	18.8
Congestive heart failure	36	0.5	56	0.5
Coronary artery disease	270	3.8	297	2.6
Chronic renal disease	44	0.6	59	0.5
Inflammatory bowel disease	26	0.4	30	0.3
Use of gastroprotective drugs	1,567	21.8	2,111	18.1
Use of warfarin	220	3.1	128	1.1
Use of antiplatelet	143	2.0	108	0.9
Use of oral steroids	963	13.4	1,356	11.7

**Table 2:** **Geometric mean of risk ratios and a summary analysis for different cohort size**, **outcome incidence and exposure prevalence of initiators of celecoxib or NSAIDs** (**ibuprofen or diclofenac**) **in a cohort 18**–**65 years old between 1 July 2003 and 30 September 2004 in the MarketScan database**

**Cohorts and variable selection methods**	**Median of exposed subjects**	**Median of exposed outcomes**	**Median of unexposed subjects**	**Median of unexposed outcomes**	Geometric mean of RR^*^	**25th**-**75th percentile of RR of samples**^**†**^	**Mean variable coverage %**^**‡**^
	**n (%)**	**n (%)**	**n (%)**	**n (%)**			
**Full cohort**^**§**^	7197 (38)	46 (0.64)	11632 (62)	71 (0.61)			
Unadjusted					1.05		
Basic covariates					0.98		
Basic and extended covariates					0.95		
Basic and hd-PS covariates					0.92		100
Basic, extended and hd-PS covariates					0.94		100
**Condition 1: 50% size sample**	3594 (38)	23 (0.64)	5821 (62)	36 (0.62)			
Unadjusted					1.02	(0.89-1.20)	
Basic covariates					0.96	(0.84-1.11)	
Basic and extended covariates					0.92	(0.80-1.09)	
Basic and hd-PS covariates					0.88	(0.74-1.07)	65
Basic, extended and hd-PS covariates					0.89	(0.74-1.11)	65
**Condition 2: 20% size sample**	1441 (38)	0 (0.66)	2325 (62)	14 (0.60)			
Unadjusted					1.10	(0.89-1.37)	
Basic covariates					1.03	(0.82-1.29)	
Basic and extended covariates					0.99	(0.79-1.24)	
Basic and hd-PS covariates					0.94	(0.71-1.21)	41
Basic, extended and hd-PS covariates					0.95	(0.70-1.25)	41
**Condition 3: 50% outcome incidence sample**	7220 (38)	23 (0.32)	11667 (62)	36 (0.31)			
Unadjusted					1.02	(0.89-1.19)	
Basic covariates					0.96	(0.84-1.13)	
Basic and extended covariates					0.93	(0.81-1.09)	
Basic and hd-PS covariates					0.90	(0.78-1.08)	65
Basic, extended and hd-PS covariates					0.91	(0.78-1.08)	65
**Condition 4: 20% outcome incidence sample**	7233 (38)	10 (0.14)	11689 (62)	14 (0.12)			
Unadjusted					1.00	(0.81-1.37)	
Basic covariates					0.94	(0.73-1.25)	
Basic and extended covariates					0.91	(0.69-1.19)	
Basic and hd-PS covariates					0.85	(0.69-1.17)	42
Basic, extended and hd-PS covariates					0.86	(0.70-1.14)	42
**Condition 5: 50% exposure prevalence sample**	3599 (19)	22 (0.61)	15230 (81)	95 (0.62)			
Unadjusted					1.02	(0.93-1.13)	
Basic covariates					0.94	(0.86-1.05)	
Basic and extended covariates					0.91	(0.83-1.02)	
Basic and hd-PS covariates					0.88	(0.79-0.98)	81
Basic, extended and hd-PS covariates					0.88	(0.79-1.00)	81
**Condition 6: 20% exposure prevalence sample**	1440 (8)	9 (0.63)	17389 (96)	108 (0.62)			
Unadjusted					0.97	(0.77-1.24)	
Basic covariates					0.89	(0.72-1.15)	
Basic and extended covariates					0.86	(0.70-1.08)	
Basic and hd-PS covariates					0.89	(0.73-1.13)	73
Basic, extended and hd-PS covariates					0.89	(0.72-1.14)	73

*Abbreviations*: basic covariates included continuous age, gender and calendar year; extended covariates included covariates adjusted for in published studies; hd-PS, high dimensional propensity score.

*Geometric mean of the risk ratio observed in 100 samples at this sampling rate.

^†^25th-75th percentiles of the risk ratio observed in 100 samples at this sampling rate.

^‡^Mean percentage of hd-PS variables in the full cohort also identified in samples.

^§^For the full cohort, all values are the number, not median.

In all cohort conditions except condition 2, where the total study size was only about 3,790, the geometric means of the hd-PS adjusted risk ratios were similar to the full cohort risk ratios. This similarity held even in cohort conditions 4 and 6, where the number of exposed patients with an outcome event was approximately 10. In all conditions except condition 6, where the exposure prevalence was only 8%, the geometric means of the hd-PS adjusted risk ratios were at least slightly closer to the RCT finding than the geometric means of the risk ratios adjusted for only the basic and extended covariates. A majority of the covariates that hd-PS identified in the full cohort were also selected by hd-PS in the samples in conditions 1, 3, and 5, where the number of exposed outcomes was at least 20, but also in condition 6, where there were only 10 exposed outcomes but a large total number of outcomes.

A scenario with combined aggregations of medications into ATC level 4 and of diagnoses into CCS level 1 consistently performed best, reducing residual confounding from 8.9% to 19.3% compared to the base scenario (Tables 3 and 4). Aggregating medications into chemical, pharmacological or therapeutic subgroups of ATC level 4, slightly improved adjusted estimates in all cohort conditions except condition 4, the 20% outcome incidence samples (data not shown). In contrast, aggregations of medications into groupings of the other ATC levels produced nearly the same or even worse adjusted risk ratios in all cohort conditions.

**Table 3:** **Risk ratios for different cohort size**, **outcome incidence and exposure prevalence of initiators of celecoxib or NSAIDs** (**ibuprofen or diclofenac**) **in a cohort 18**–**65 years old between 1 July 2003 and 30 September 2004 in the MarketScan database by using the High**-**Dimensional Propensity Score** (**hd**-**PS**) **adjustment with different aggregation methods**

**Cohort and variable selection method**		**Base scenario**	**Medications**						**Medical diagnoses**								**Combined**
			**No Rx**	ATC* Level					**No Dx**	**CCS**^**† **^**Level**					**ICD-9**^**‡**^		**ATC 4th + CCS 1st**
				**1st**	**2nd**	**3rd**	**4th**	**5th**		**1st**	**2nd**	**3rd**	**4th**	**Universal**	**3-digit**	**4-digit**	
Unadjusted		1.05															
Basic covariates		0.98															
Basic and extended covariates		0.95															
Basic and hd-PS covariates		0.92	0.94	0.93	0.92	0.92	0.90	0.91	0.88	0.90	0.89	0.92	0.92	0.94	0.95	0.94	0.85
	%^§^		3.9	2.6	0.0	0.8	−2.9	−1.4	−7.0	−3.7	−4.4	0.10	1.0	3.6	5.1	4.1	−12.1
Basic, extended and hd PS covariates		0.94	0.91	0.96	0.94	0.94	0.90	0.93	0.91	0.91	0.92	0.95	0.94	0.96	0.96	0.95	0.88
	%^§^		−5.0	3.7	−0.5	−0.7	−6.0	−1.3	−5.0	−4.4	−2.5	1.0	0.6	3.6	4.0	2.1	−10.9
hd-PS covariates (k = 500)^║^																	
Outpatient diagnoses (n)		136	224	198	177	154	144	133	0	32	90	97	54	123	133	139	34
Inpatient diagnoses (n)		9	12	11	11	9	9	7	0	22	18	19	5	16	14	11	23
Medication (n)		167	0	36	76	122	148	177	247	216	186	181	213	171	166	163	194
Outpatient procedures (n)		152	220	211	194	174	161	148	210	188	166	163	187	153	151	151	206
Inpatient procedures (n)		36	44	44	42	41	38	35	43	42	40	40	41	37	36	36	43

*Abbreviations*: basic covariates included continuous age, gender and calendar year; extended covariates included covariates adjusted for in published studies; hd-PS, high dimensional propensity score. Base scenario used up to 5-digit ICD-9, procedures, generic drugs for five data dimensions of the hd-PS.

No Rx: the scenario using up to 5-digit ICD-9 and procedures for 4 data dimensions of the hd-PS.

No Dx: scenario using procedures and generic drugs for 3 data dimensions of the hd-PS.

^*^ATC: 5 levels of the Anatomical Therapeutic Chemical classification.

^†^CCS: four levels of the Clinical Classification Software; Universal, the most granular CCS code available for each ICD-9 code.

^‡^ ICD-9: International Classification of Diseases, 9th Revision, Clinical Modification.

^§^: % proportional difference in absolute degree of estimated confounded between estimates for the specific aggregation scenario and the basic scenario at the same variable selection method on the natural log scale with RCT finding of 0.5. The presumptive amount of confounding in the basic scenario A = │ln(adjusted RR) – ln(0.5)│; in each aggregation method B = │ln(adjusted RR) – ln(0.5)│; and C = B/A –1.

^║^: number of hd-PS covariates retained in the final propensity score model. Total (k=500) and from each data dimension (n).

**Table 4:** Geometric mean of risk ratios for different cohort size, outcome incidence and exposure prevalence of initiators of celecoxib or NSAIDs (ibuprofen or diclofenac) in a cohort 18–65 years old between 1 July 2003 and 30 September 2004 in the MarketScan database by using the High-Dimensional Propensity Score (hd-PS) adjustment with different aggregation scenarios

**Cohort and confounding adjustment method**	**Base scenario**	Combined ATC^*^4th level and CCS^**† **^**1st level**	**% Proportional difference**^**‡**^
**Condition 1: 50% size sample**			
Unadjusted	1.02		
Basic and hd-PS covariates	0.88	0.83	−9.9%
Basic, extended and hd-PS covariates	0.89	0.84	−8.9%
**Condition 2: 20% size sample**			
Unadjusted	1.10		
Basic and hd-PS covariates	0.94	0.87	−12.0%
Basic, extended and hd-PS covariates	0.95	0.88	−11.9%
**Condition 3: 50% outcome incidence sample**			
Unadjusted	1.02		
Basic and hd-PS covariates	0.90	0.84	−11.9%
Basic, extended and hd-PS covariates	0.91	0.85	−11.3%
**Condition 4: 20% outcome incidence sample**			
Unadjusted	1.00		
Basic and hd-PS covariates	0.85	0.81	−10.4%
Basic, extended and hd-PS covariates	0.86	0.82	−9.8%
**Condition 5: 50% exposure prevalence sample**			
Unadjusted	1.02		
Basic and hd-PS covariates	0.88	0.81	−14.4%
Basic, extended and hd-PS covariates	0.88	0.82	−12.7%
**Condition 6: 20% exposure prevalence sample**			
Unadjusted	0.97		
Basic and hd-PS covariates	0.89	0.79	−19.3%
Basic, extended and hd-PS covariates	0.89	0.81	−16.3%

Abbreviations: basic covariates included continuous age, gender and calendar year; extended covariates included covariates adjusted for in published studies; hd-PS, high dimensional propensity score.

Base scenario used up to 5-digit ICD-9, procedures, generic drugs for five data dimensions of the hd-PS.

^*^ATC: 5 levels of the Anatomical Therapeutic Chemical classification.

^†^CCS: Four levels of the Clinical Classification Software; Universal, the most granular CCS code available for each ICD-9 code.

^‡^: % proportional difference in absolute degree of estimated confounded between estimates for the specific aggregation scenario and the basic scenario at the same variable selection method on the natural log scale with RCT finding of 0.50. The presumptive amount of confounding in the basic scenario A = │ln(adjusted RR) – ln(0.5)│; in each aggregation method B = │ln(adjusted RR) – ln(0.5)│; and C = B/A – 1.

When we experimented with different aggregations for diagnoses, without any aggregation for medications, aggregating ICD-9 diagnosis codes into different CCS levels inconsistently changed the adjusted risk ratios. Note that in our empirical setting, not controlling for any measure of diagnoses resulted in the estimate closest to the RCT finding (RRs in column “No Dx” of Table 3). When we aggregated ICD-9 diagnosis codes into CCS levels 1 or 2, the adjusted risk ratios in the samples were generally closer to the RCT finding. In contrast, aggregations of ICD-9 codes into CCS universal, CCS level 3, CCS level 4, or 3- or 4-digit ICD-9 groupings did not improve the adjusted point estimates (data not shown).

## Discussion

We hypothesized that aggregations of medications and medical diagnoses into certain levels of ATC or CCS would help the performance of the hd-PS, especially with smaller cohort size, rarer outcome incidence or lower exposure prevalence. To explore these hypotheses, we selected a retrospective cohort where, as has been previously observed, the hd-PS adjustment for confounding yielded an adjusted RR slightly closer to the RCT findings [21-26] than did PS adjustment using a limited number of investigator predefined covariates [16,18].

Of the 500 covariates identified by hd-PS in the full cohort, most were also identified by hd-PS in the random samples with fewer observations, rarer outcomes, or lower prevalence of treatments. Aggregations of medications into ATC level 4 alone or in combination with aggregation of diagnoses into CCS level 1 improved the hd-PS adjustment for confounding in the full cohort and most of the samples. The strength of our results on the effect of aggregating diagnoses is limited, however, by the fact that the overall confounding by co-morbidity was attenuated in the presence of 500 hd-PS covariates from medications, outpatient procedures and inpatient procedures in our empirical setting.

In general, aggregation of potential covariates into higher-level groupings increases the number of covariates that are present in at least 100 observations (the default requirement of the hd-PS version 1) and increases the prevalence of the covariate in exposed and unexposed groups which increases the covariate’s prioritization from the Bross formula if it is associated with treatment [19]. But aggregation may simultaneously weaken covariate-exposure and/or covariate-outcome relations, reducing prioritization in the Bross formula [19]. The latter also has the potential to change the impact of control for the aggregated covariate on the adjusted risk ratios. The hd-PS algorithm theoretically may not favor the aggregation of confounder information. However, in particular cases (e.g., small samples, rare outcome incidence and low exposure prevalence), aggregations potentially help the hd-PS to reduce residual bias, for example, in this study. Version 2 of the hd-PS algorithm, which removed the restriction of a minimum 100 occurrences per potential confounder, allows important confounders to have a higher chance for the variable selection process and may improve bias reduction for treatment effect in small sample sizes and low exposure prevalence.

Grouping medications into ATC level 4 instead of the original generic drugs helped the hd-PS to robustly function in the samples, except for the 20% outcome incidence (condition 4). The use of other ATC levels for aggregating medications did not provide benefit and even resulted in some harm. For example, ATC level 4 code B01AC (platelet aggregation inhibitors excluding heparin) includes the following level 5 codes: B01AC04 (clopidrogel), B01AC05 (ticlopidine), B01AC07 (dipyridamole), B01AC23 (cilostazol), and B01AC30 (combined drugs). The latter four codes each occurred in fewer than the 100 observation minimum that hd-PS requires by default and so would not be eligible for inclusion in the hd-PS adjustment. With ATC level 5 for medications, the hd-PS algorithm selected code B01AC04 (frequency 218, covariate-exposure RR = 1.5, covariate-outcome RR = 3.8 – Table 5). Using ATC level 4 for medications, the hd-PS selected ATC level 4 code B01AC which had a slightly higher frequency (253), the same covariate-exposure (RR = 1.5) but slightly weaker covariate-outcome (RR = 3.3) associations. Situations like this may account for the observed improvement in confounding control in the ATC level 4 aggregation (e.g., RR of 0.83 in 20% exposure prevalence scenario) compared with scenarios that used ATC level 5 (e.g., RR of 0.88). Additional examples to illustrate the changes in prevalence, covariate-exposure and covariate-outcome relations from aggregation of clopidrogel and warfarin from level 5 to ATC levels 4, 3, 2 and 1 are in Table 5. The ATC level 4 with pharmacological subgroups seems the most appropriate level for aggregation of medications in this study.

**Table 5:** Changes of prevalence, covariate-exposure and covariate-outcome relations when we aggregated potential confounders, clopidrogel and warfarin from level 5 to levels 4, 3, 2 and 1 of the Anatomical Therapeutic Chemical (ATC) classification

**Dictionary/Level**	**Code**	**Description**	**Frequency**	**Frequency type**	**Covariate exposure risk ratio**	**Covariate-outcome risk ratio**	**Prevalence in both groups**	**Included in lower level**
Generic drug		Clopidrogrel	218	once	1.5	3.8	0.012	
Generic drug		Clopidrogrel	218	sporadic	1.4	2.9	0.012	
Generic drug		Warfarin	319	once	1.6	2.0	0.017	
Generic drug		Warfarin	319	sporadic	1.7	1.3	0.017	
ATC level 5	B01AC04	Clopidrogrel	218	once	1.5	3.8	0.012	
ATC level 5	B01AC04	Clopidrogrel	218	sporadic	1.4	2.9	0.012	
ATC level 5	B01AA03	Warfarin	319	once	1.6	2.0	0.017	
ATC level 5	B01AA03	Warfarin	319	sporadic	1.7	1.3	0.017	
ATC level 4	B01AC	Platelet aggregation inhibitors excluding heparin	253	once	1.5	3.3	0.013	
ATC level 4	B01AC	Platelet aggregation inhibitors excluding heparin	253	sporadic	1.5	2.5	0.013	
	B01AC04	Clopidrogrel	218					Yes
	B01AC05	Ticlopidine	1				0.000	No
	B01AC07	Dipyridamole	6				0.000	No
	B01AC23	Cilostazol	25				0.000	No
	B01AC30	Combinations	11				0.000	No
ATC level 4	B01AA	Vitamin K antagonists	319	once	1.6	2.0	0.017	
ATC level 4	B01AA	Vitamin K antagonists	319	sporadic	1.7	1.3	0.017	
ATC level 3	B01AA03	Warfarin	319					Yes
ATC level 3	B01A	Antithrombotic agents	637	once	1.5	1.5	0.034	
ATC level 2	B01A	Antithrombotic agents	637	sporadic	1.6	2.0	0.034	
ATC level 2	B01	Antithrombotic agents	637	once	1.5	1.5	0.034	
ATC level 2	B01	Antithrombotic agents	637	sporadic	1.6	2.0	0.034	
ATC level 1	B	Blood and blood forming organs	1049	once	1.4	1.4	0.056	
ATC level 1	B	Blood and blood forming organs	1049	sporadic	1.4	1.9	0.056	
ATC level 1	B	Blood and blood forming organs	1049	frequent	1.5	2.0	0.025	

As for diagnostic codes, ICD-9 code 530.1 includes 530.11 (reflux esophagitis) and the additional codes 530.10 (esophagitis unspecified), 530.12 (acute esophagitis) and 530.19 (other esophagitis). In our study, the latter three codes each occurred in fewer than the 100 observation minimum that hd-PS requires by default and so would not be eligible for inclusion in the PS adjustment. With 5-digit granularity for diagnoses, the hd-PS selected ICD-9 code 530.11 (frequency 165, covariate-exposure RR = 1.3, covariate-outcome RR = 5.0 – see, Additional file 1: Table S6). Using 4-digit granularity for diagnoses, the hd-PS selected ICD-9 code 530.1 (esophagitis) which had a higher frequency (217) but slightly weaker covariate-exposure (RR = 1.2) and covariate-outcome (RR = 4.6) associations. Situations like this could account for the slight worsening of confounding control in the 4-digit ICD-9 aggregation compared with the base case (up to 5-digit ICD-9). Additional examples to illustrate the changes in prevalence, covariate-exposure and covariate-outcome relations when we aggregated potential confounders, ICD-9 codes 530.11 (reflux esophagitis) and 530.81 (esophageal reflux) from 5-digit ICD-9 into 4-, 3-digit ICD-9, and CCS levels 4, 3, 2 and 1 are in Additional file 1: Table S6. It is worth noting that not all ICD-9 diagnosis codes have their equivalent CCS codes in all 4 levels [32]. This issue was more pronounced in CCS levels 3 and 4. Using the most granular CCS code available for each ICD-9 code in the universal CCS did not improve results in most samples and the full cohort. We also did not observe any benefit while aggregating ICD-9 codes into first 3- or 4-digit groupings [16,31]. Since CCS has only 18 groupings for level 1 and 134 groupings for level 2, it could be argued that the benefit from aggregation comes about by enabling more variables from the other data dimensions (medications, inpatient and outpatient procedures) to fit within the 500 variable maximum in the hd-PS default. To address this concern, we also experimented with a maximum of k = 3,000 variables and consistently observed the benefit of aggregation of ICD-9 into CCS levels 1 or 2. Similarly, ATC level 1 has 14 groups, whereas level 4 has over 800 groupings, but aggregation of medications into ATC level 4 outperformed aggregation into level 1.

Our study has several limitations. This study empirically compared estimates from different aggregations and assumed any treatment effect estimates closer to the RCT findings to be less biased by confounding. There was relatively little confounding present in the data, and the effect estimates did not change much after adjustment for the baseline covariates. The magnitude of the percentage reductions in confounding depends on the value selected as the unconfounded value; however, the precise value selected from the published RCT [21-26] does not affect the ranking of performance across scenarios. Our comparison relies on the assumption that the codes in the original database are accurate. Also, our study is based in a single cohort in which hd-PS performed reasonably well. Fully specified simulations with true risk ratios in diversified scenarios could be used to prove the advantage of aggregation under certain conditions but would be unable to answer the important question of magnitude in real world settings. It is nevertheless unclear whether our findings regarding the effects of aggregation of medications and diagnostic codes on the performance of the hd‒PS algorithm apply to other treatment‒outcome pairs that may be subject to confounding by different factors. Studies with few events or small size may suffer from small sample bias or overfit PS models and outcome models using PS deciles to estimate adjusted risk ratios [36,37]. The small number of UGI complication cases produced imprecise estimates and insufficient power to confirm differences between the different methods. The computer time requirements of the hd-PS algorithm constrained our ability to increase the size of our samples beyond 100 for each cohort condition. We thus should compare results with and without aggregations within each condition, but not across conditions. However, we are interested in bias which pertains to expected values of point estimates and statistical significance plays no defensibly useful role in the assessment or measurement of bias. Moreover, each aggregation scenario had six cohort conditions (600 samples). Thus, consistent patterns (the combined ATC level 4 plus CCS level 1) are supported by a large number of samples. Users of the hd-PS methodology should screen and remove instrumental variables and collider bias candidates [10-12]. This topic is out of the scope of this study.

Further studies may explore examples of no drug effect on the outcome, increased drug-outcome risk, more common outcomes, and compare the aggregation approaches with the zero-cell correction or exposure-based association selection for the hd-PS [38], develop appropriate methods to replace missing codes in CCS levels, appropriate aggregations for procedures, simultaneous aggregation of diagnoses, medications and procedures, evaluation of the hd-PS functions in cohorts with different cohort size, outcome incidence and exposure prevalence.

## Conclusion

Aggregation of drug and diagnostic codes using hierarchical coding systems may improve the performance of the hd-PS to control for confounders in cohorts with small size, low outcome incidence or low exposure prevalence but the balance of advantages and disadvantages of aggregation is likely to vary across settings.

## Competing interests

All authors declare that they have no competing interests.

## Authors’ contributions

HVL, under the supervision of his advisor TS, conceived research questions, developed study design and methods, carried out statistical analysis, interpreted results and drafted the manuscript. CP, MAB, VJS and KJB, who were dissertation committee members, advised on the study design, methods, statistical analyses and manuscript. JBL was responsible for interpretation of results and manuscript preparation. All authors commented on successive drafts, and read and approved the final manuscript.

## Pre-publication history

The pre-publication history for this paper can be accessed here:

http://www.biomedcentral.com/1471-2288/13/142/prepub

## Supplementary Material

Additional file 1: Table S6 Changes of prevalence, covariate-exposure and covariate-outcome relations when we aggregated potential confounders, ICD-9 codes 53011 (reflux esophagitis) and 53081 (esophageal reflux) from 5-digit ICD-9 into 4-, 3-digit ICD-9, and levels 4, 3, 2 and 1 of the Clinical Classification Software (CCS). Click here for file

## References

[B1] Davis RL Kolczak M Lewis E Nordin J Goodman M Shay DK Platt R Black S Shinefield H Chen RT Active surveillance of vaccine safety: a system to detect early signs of adverse events Epidemiology 2005 16 3 336 341 10.1097/01.ede.0000155506.05636.a4 15824549

[B2] Brown JS Kulldorff M Chan KA Davis RL Graham D Pettus PT Andrade SE Raebel MA Herrinton L Roblin D Boudreau D Smith D Gurwitz JH Gunter MJ Platt R Early detection of adverse drug events within population-based health networks: application of sequential testing methods Pharmacoepidemiol Drug Saf 2007 16 12 1275 1284 10.1002/pds.1509 17955500

[B3] Lieu TA Kulldorff M Davis RL Lewis EM Weintraub E Yih K Yin R Brown JS Platt R Vaccine Safety Datalink Rapid Cycle Analysis Team Real-Time vaccine safety surveillance for the early detection of adverse events Med Care 2007 45 10 S89 S95 17909389 10.1097/MLR.0b013e3180616c0a

[B4] Rubin DB Estimating causal effects from large data sets using the propensity score Ann Intern Med 1997 127 757 763 10.7326/0003-4819-127-8_Part_2-199710151-00064 9382394

[B5] Stürmer T Schneeweiss S Brookhart MA Rothman KJ Avorn J Glynn RJ Analytic strategies to adjust confounding using exposure propensity scores and disease risk scores: nonsteroidal antiinflammatory drugs and short-term mortality in the elderly Am J Epidemiol 2005 161 9 891 898 10.1093/aje/kwi106 15840622 PMC1407370

[B6] Stürmer T Joshi M Glynn RJ Avorn J Rothman KJ Schneeweiss S A review of applications of propensity score methods showed increased use but infrequently different estimates compared with other methods J Clin Epidemiol 2006 59 437 447 16632131 10.1016/j.jclinepi.2005.07.004 PMC1448214

[B7] Glynn RJ Schneeweiss S Stürmer T Indications for propensity scores and review of their use in pharmacoepidemiology Basic Clin Pharmacol Toxicol 2006 98 253 259 10.1111/j.1742-7843.2006.pto_293.x 16611199 PMC1790968

[B8] Perkins SM Tu W Underhill MG Zhou XH Murray MD The use of propensity scores in pharmacoepidemiologic research Phamacoepidemiol Drug Saf 2000 9 93 101 10.1002/(SICI)1099-1557(200003/04)9:2<93::AID-PDS474>3.0.CO;2-I 19025807

[B9] Robins JM Mark SD Newey WK Estimating exposure effects by modeling the expectation of exposure conditional on confounders Biometrics 1992 48 479 495 10.2307/2532304 1637973

[B10] Rubin DB On principles for modeling propensity score in medical research Phamacoepidemiol Drug Saf 2005 14 227 238 10.1002/pds.986 15386710

[B11] Brookhart MA Schneeweiss S Rothman KJ Glynn RJ Avorn J Stürmer T Variable selection for propensity score models Am J Epidemiol 2006 163 12 1149 1156 10.1093/aje/kwj149 16624967 PMC1513192

[B12] Greenland S Quantifying biases in causal models: classical confounding vs. collider-stratification bias Epidemiology 2003 14 3 300 306 12859030

[B13] Brookhart MA Stürmer T Glynn RJ Rassen J Schneeweiss S Confounding control in healthcare database research: challenges and potential approaches Med Care 2010 48 6 S114 S120 20473199 10.1097/MLR.0b013e3181dbebe3 PMC4024462

[B14] Seeger JD Kurth T Walker AM Use of propensity score technique to account for exposure-related covariates: an example and lesson Med Care 2007 45 S143 S148 10.1097/MLR.0b013e318074ce79 17909373

[B15] Johannes CB Koro CE Quinn SG Cutone JA Seeger JD The risk of coronary heart disease in type 2 diabetic patients exposed to thiazolidinediones compared to metformin and sulfonylurea therapy Pharmacoepidemiol Drug Saf 2007 16 504 512 10.1002/pds.1356 17245800

[B16] Schneeweiss S Rassen JA Glynn RJ Avorn J Mogun H Brookhart MA High-dimensional propensity score adjustment in studies of treatment effects using health care claims data Epidemiology 2009 20 4 512 522 10.1097/EDE.0b013e3181a663cc 19487948 PMC3077219

[B17] Le VH Beach JK Powell G Pattishall E Ryan P Mera RM Performance of a semi-automated approach for risk estimation using a common data model for longitudinal healthcare databases Stat Methods Med Res 2013 22 1 97 112 10.1177/0962280211403599 21680614

[B18] Toh S Rodríguez AGL Hernán AM Confounding adjustment via a semi‒automated high‒dimensional propensity score algorithm: an application to electronic medical records Pharmacoepidemiology and drug safety 2011 20 849 857 10.1002/pds.2152 21717528 PMC3222935

[B19] Bross ID Spurious effects from an extraneous variable J Chronic Dis 1966 19 6 637 647 10.1016/0021-9681(66)90062-2 5966011

[B20] Brookhart MA Wang PS Solomon DH Schneeweiss S Evaluating short-term drug effects using a physician-specific prescribing preference as an instrumental variable Epidemiology 2006 17 3 268 275 10.1097/01.ede.0000193606.58671.c5 16617275 PMC2715942

[B21] Silverstein FE Faich G Goldstein JL Simon LS Pincus T Whelton A Makuch R Eisen G Agrawal NM Stenson WF Burr AM Zhao WW Kent JD Lefkowith JB Verburg KM Geis GS Gastrointestinal toxicity with celecoxib vs nonsteroidal anti-inflammatory drugs for osteoarthritis and rheumatoid arthritis: the CLASS study: A randomized controlled trial Celecoxib Long-term Arthritis Safety Study JAMA 2000 284 10 1247 1255 10.1001/jama.284.10.1247 10979111

[B22] Singh G Fort JG Goldstein JL Levy RA Hanrahan PS Bello AE Andrade-Ortega L Wallemark C Agrawal NM Eisen GM Stenson WF Triadafilopoulos G; SUCCESS-I Investigators Celecoxib versus naproxen and diclofenac in osteoarthritis patients: SUCCESS-I Study Am J Med 2006 119 3 255 266 10.1016/j.amjmed.2005.09.054 16490472

[B23] Deeks JJ Smith LA Bradley MD Efficacy, tolerability, and upper gastrointestinal safety of celecoxib for treatment of osteoarthritis and rheumatoid arthritis: systematic review of randomised controlled trials BMJ 2002 325 7365 619 10.1136/bmj.325.7365.619 12242171 PMC126301

[B24] Moore RA Derry S Makinson GT McQuay HJ Tolerability and adverse events in clinical trials of celecoxib in osteoarthritis and rheumatoid arthritis: systematic review and meta-analysis of information from company clinical trial reports Arthritis Res Ther 2005 7 3 R644 R665 10.1186/ar1704 15899051 PMC1174947

[B25] Goldstein JL Silverstein FE Agrawal NM Hubbard RC Kaiser J Maurath CJ Verburg KM Geis GS Reduced risk of upper gastrointestinal ulcer complications with celecoxib, a novel COX-2 inhibitor Am J Gastroenterol 2000 95 7 1681 1690 10.1111/j.1572-0241.2000.02194.x 10925968

[B26] Goldstein JL Significant upper gastrointestinal events associated with conventional NSAID versus celecoxib J Rheumatol Suppl 2000 60 25 28 11032099

[B27] Ray WA Evaluating medication effects outside of clinical trials: new-user designs Am J Epidemiol 2003 158 9 915 920 10.1093/aje/kwg231 14585769

[B28] MarketScan^®^ Commercial Claims and Encounters of Thomson Reuters Healthcare 2011 [http://thomsonreuters.com/products_services/healthcare/healthcare_products/a-z/marketscan_research_analytics/]

[B29] Raiford DS Perez Gutthann S Garcia Rodriguez LA Positive predictive value of ICD-9 codes in the identification of cases of complicated peptic ulcer disease in the Saskatchewan hospital automated database Epidemiology 1996 7 101 104 10.1097/00001648-199601000-00018 8664388

[B30] The Anatomical Therapeutic Chemical (ATC) classification 2012 [http://www.whocc.no/] 10.1371/journal.pone.0035254 PMC3325992 22514724

[B31] Hoskins H Jr Hildebrand P Lum F The American Academy of Ophthalmology Adopts SNOMED CT as Its Official Clinical Terminology Ophthalmology 2008 115 2 225 226 10.1016/j.ophtha.2007.11.026 18243903

[B32] Clinical Classifications Software (CCS) for ICD-9-CM 2012 [http://www.hcup-us.ahrq.gov/toolssoftware/ccs/ccs.jsp]

[B33] García Rodríguez LA Jick H Risk of upper gastrointestinal bleeding and perforation associated with individual non‒steroidal anti‒inflammatory drugs Lancet 1994 343 769 772 10.1016/S0140-6736(94)91843-0 7907735

[B34] Gutthann SP García Rodríguez LA Raiford DS Individual nonsteroidal antiinflammatory drugs and other risk factors for upper gastrointestinal bleeding and perforation Epidemiology 1997 8 18 24 10.1097/00001648-199701000-00003 9116088

[B35] Hernández‒Díaz S García Rodríguez LA Association between nonsteroidal anti‒inflammatory drugs and upper gastrointestinal tract bleeding/perforation: an overview of epidemiologic studies published in the 1990s Arch Intern Med 2000 160 2093 2099 10.1001/archinte.160.14.2093 10904451

[B36] Greenland S Schwartzbaum JA Finkle WD Problems due to small samples and sparse data in conditional logistic regression analysis Am J Epidemiol 2000 151 5 531 539 10.1093/oxfordjournals.aje.a010240 10707923

[B37] Peduzzi P Concato J Kemper E Holford TR Feinstein AR A simulation study of the number of events per variable in logistic regression analysis J Clin Epidemiol 1996 49 12 1373 1379 10.1016/S0895-4356(96)00236-3 8970487

[B38] Rassen JA Glynn JR Brookhart MA Schneeweiss S Covariate selection in high-dimensional propensity score analyses of treatment effects in small samples Am J Epidemiol 2011 173 12 1404 1413 10.1093/aje/kwr001 21602301 PMC3145392

