# Supplementary material for: Effects of aggregation of drug and diagnostic codes on the performance of the high-dimensional propensity score algorithm: an empirical example
Source: BMC Med Res Methodol. 2013 Nov 19;13:142. doi: 10.1186/1471-2288-13-142 (PMC3840608; doi:10.1186/1471-2288-13-142)
Supplement: Additional file 1: Table S6 — Changes of prevalence, covariate-exposure and covariate-outcome relations when we aggregated potential confounders, ICD-9 codes 53011 (reflux esophagitis) and 53081 (esophageal reflux) from 5-digit ICD-9 into 4-, 3-digit ICD-9, and levels 4, 3, 2 and 1 of the Clinical Classification Software (CCS). [file 1471-2288-13-142-S1.docx]

**Additional file 1**

**Table 6.** Changes of prevalence, covariate-exposure and covariate-outcome relations when we aggregated potential confounders, ICD-9 codes 53011 (reflux esophagitis) and 53081 (esophageal reflux) from 5-digit ICD-9 into 4-, 3-digit ICD-9, and levels 4, 3, 2 and 1 of the Clinical Classification Software (CCS)
